# Scar-like lesion on dorsal nose (cellular neurothekeoma)

**DOI:** 10.1186/1746-160X-3-39

**Published:** 2007-11-30

**Authors:** Larissa Dorina López-Cepeda, Gisela Navarrete-Franco, Josefa Novales-Santacoloma, Julio Enriquez-Merino

**Affiliations:** 1 Consultation, Centro Dermatológico "Dr. Ladislao de la Pascua", Mexico city, Mexico; 2 Dermatopathology Department, Centro Dermatológico "Dr. Ladislao de la Pascua", Mexico city, Mexico; 3 Dermatopathology Department, Centro Dermatológico "Dr. Ladislao de la Pascua", Mexico city, Mexico; 4 Surgery Department, Centro Dermatológico "Dr. Ladislao de la Pascua", Mexico city, Mexico

## Abstract

Neurothekeomas are tumors of neural differentiation and of unknown origin that occur in females at the 2^nd ^and 3^rd ^decades of life. They usually affect the face with an unspecific clinical aspect. The histological features include cellular or mixoid differentiation and immunohistochemistry can be positive for protein s-100, vimentin and epithelilal membrane antigen (EMA).

This case report presents a 13-year-old female patient with nasal neurothekeoma of cellular variety and strongly positive for vimentin and s-100; and negative for EMA.

## Background

Neurothekeoma (NK) is a tumor of neural differentiation first described by Harkin and Reed in 1969 [1] and later termed "neurothekeoma" by Gallager and Helwig in 1980 [2]. Being extremely rare (1 of 4000 biopsies in international reports) [1], it is hitologically constituted by cellular proliferation arranged in lobules or fascicles immersed in an amorphous matrix in the dermis and rarely in subcutaneous tissue [3].

The origin of this tumor is unknown. Some authors consider it as Schwann cells, [4] while others state that perineural tissue or supporting structures of peripheric nerves, fibroblasts, and muscle could be responsible [4]. It has even been considered as a variant of the following dermatoses: dermatofibroma [5], pilar epithelioid leiomioma [6], plexiform neurofibroma, [7] and nerve sheath myxoma [8].

NK presents frequently in females between 10–66 years (especially among the 2^nd ^and 3^rd ^decades of life). It is most commonly located is on the head, face and superior mid-body. To date, 21 reports have shown facial occurrence (with one patient revealing nasal manifestation) and 31 reports demonstrate occurrence of NK in patients younger than 20 years old [5,9-11]. Other reports have documented different locations of occurrence including tongue [9], oral mucosa, eyelids [10], trunk (dorsum and shoulders) and superior extremities (superior mid body).

NK has an unspecific clinical aspect, more frequently presenting as a cupuliform, firm, flesh-colored or hyperpigmented papule-like formation which rarely exceeds 10 mm with capillaries on its surface and may be asymptomatic. The evolution varies between 18 months and 30 years; with a slowly growing, generally benign course.

Diagnosis can be made by conventional histopathology [1,2,4] yielding two basic patterns:

1. Mucinous or myxoid: It is the most frequent form. It has an evident fascicular and tabicated aspect with abundant mucinous and amorphous substance, composed principally of acid mucopolysaccharides.

2. Cellular – epitheloid or fusiform cells with a cellular pattern and fascicle-nodular aggregation, circumscribed to reticular dermis, showing a grenz zone separating the dermis from the subacent tumor. There can be some mitotic figures [12], atypical features [13] and metachromasia with Giemsa stain [14]. Collagen may be sclerotic with some lymphocytic infiltration. Barnhill, et al. [13] consider that the cellular neurothekeoma might be an early form (more frequent in young patients), whilethe mucinous type might represent a tumor of longer evolution (frequent in older patients).

NK is positive with alcian blue at pH 2.5, and there are reports of metachromasia with Giemsa stain [1].

By immunohistochemistry, protein S-100 [14] has been found positive, as well as other stains: epithelilal membrane antigen (EMA), vimentin and neuron specific enolase (NSE), (with controversial results) [15]. Other useful markers are: myelin basic protein, neurofilaments, glial fibrillary acidic protein (GFAP), keratin and Leu-7 [11], NK1/C3 and PGP9.5 [2,16].

Electronic microscopy is not useful for diagnosis. Differential diagnosis should be made chiefly with scars, dermatofibromas, nevi, lymphocytomas and adnexae tumors among others [2,4,12,13].

## Case presentation

**Figure 1:**
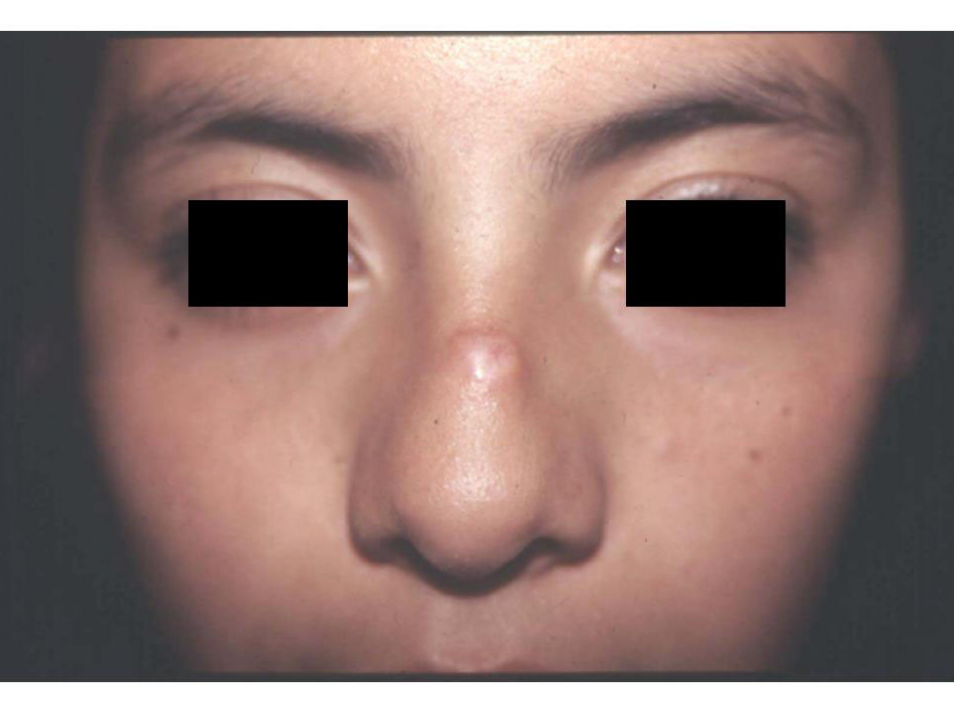
A 0.3 cm, soft, light pink neoformation in the dorsal aspect of nose.

**Figure 2:**
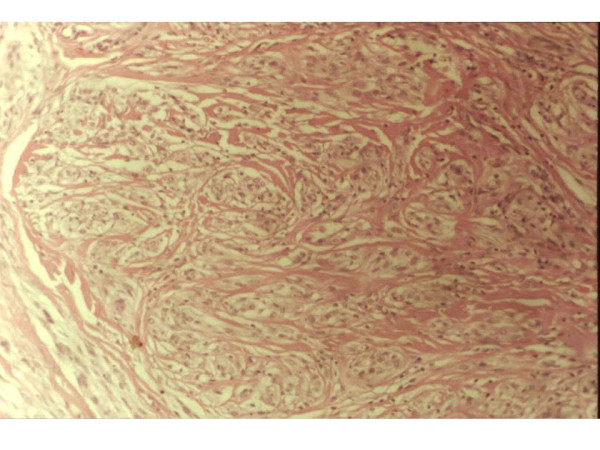
H-E 40× – Dense infiltrates of fusiform cells arranged in nests, with some mitoses (pleomorphic cytology).

A 13-year-old female referred to the clinic with dermatosis at the dorsal aspect of the nose. The lesion was 0.3 cm in diameter, flat, soft, swollen; and light pink in color, with superficial telangiectasias. Reportedly the lesion had appeared 6 months ago, and had remained asymptomatic (figure 1). The patient history was non-contributory, including the absence of prior trauma. The incisional skin biopsy showed atrophic epidermis with lax hyperkeratosis, sub-papillar moderate lymphocytic infiltrate; dense dermal infiltrate of fusiform cells arranged in nests with mitoses and hypotrophic adnexae. The collagen surrounding the neoformation had a normal aspect (figures 2). Mucin stain (Mucicarmin of Mayer) and neurofilament stains (Bielchowsky) were positive [17].

Immunohistochemistry was strongly positive for vimentin, lightly postitive for s-100, negative for EMA and positive for mucin. Based on biopsy and immunohistochemistry, the diagnosis of Neurothekeoma was made.

**Figure 3:**
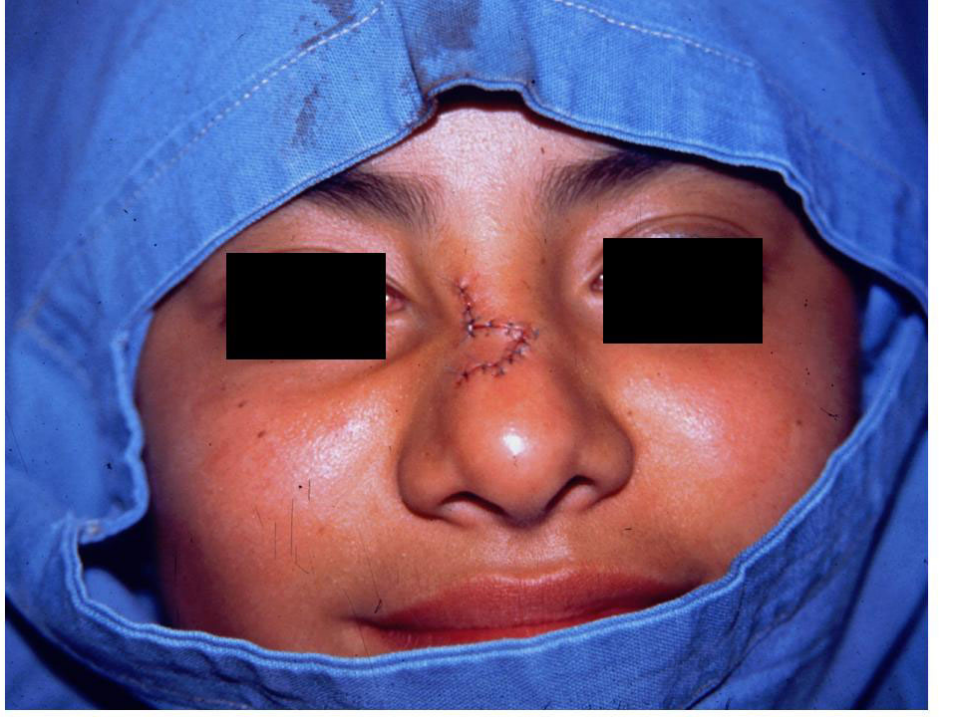
Defect correction with a Limbert flap.

The treatment included complete extirpation of the lesion, followed by esthetic correction with a Limbert flap (figure 3).

## Conclusion

In the present case, clinical diagnosis of neurothekeoma was difficult to make since the lesion had a scar-like pattern, without previous history of trauma. Histological examination is definetely essential for the diagnosis, and must be interpreted by experienced dermatopathologists. An interesting feature of this case is that it was positive for both of the neurofilament stains (Bielchowsky) and mucin stain (Mucicarmin of Mayer) showing its dual differentiation: neural and myxoid.

## Authors' contributions

All author(s) read and approved the final manuscript.

LDLC, made the clinical diagnosis, follow up of the patient, surgical treatment and writing the manuscript.

GNF and JNS, made the histological diagnosis.

JEM, planned and assisted the patient's surgical treatment.
